# Management of relapse in acute promyelocytic leukaemia treated with up‐front arsenic trioxide‐based regimens

**DOI:** 10.1111/bjh.17221

**Published:** 2020-11-20

**Authors:** N. A. Fouzia, Vibhor Sharma, Saravanan Ganesan, Hamenth K. Palani, Nithya Balasundaram, Sachin David, Uday P. Kulkarni, Anu Korula, Anup J. Devasia, Sukesh C. Nair, Nancy Beryl Janet, Aby Abraham, Thenmozhi Mani, Jeyaseelan Lakshmanan, Poonkuzhali Balasubramanian, Biju George, Vikram Mathews

**Affiliations:** ^1^ Department of Haematology Christian Medical College Vellore India; ^2^ Department of Immunohaematology and Transfusion Medicine Christian Medical College Vellore India; ^3^ Department of Biostatistics Christian Medical College Vellore India

**Keywords:** relapse acute promyelocytic leukaemia, arsenic trioxide (ATO), post up‐front ATO relapse, autologous stem cell transplant

## Abstract

The standard of care for patients with acute promyelocytic leukaemia (APL) relapsing after front‐line treatment with arsenic trioxide (ATO)‐based regimens remains to be defined. A total of 67 patients who relapsed after receiving ATO‐based up‐front therapy and were also salvaged using an ATO‐based regimen were evaluated. The median (range) age of patients was 28 (4–54) years. While 63/67 (94%) achieved a second molecular remission (MR) after salvage therapy, three (4·5%) died during salvage therapy. An autologous stem cell transplant (auto‐SCT) was offered to all patients who achieved MR, 35/63 (55·6%) opted for auto‐SCT the rest were administered an ATO + all‐*trans* retinoic acid maintenance regimen. The mean (SD) 5‐year Kaplan–Meier estimate of overall survival and event‐free survival of those who received auto‐SCT *versus* those who did not was 90·3 (5·3)% *versus* 58·6 (10·4)% (*P* = 0·004), and 87·1 (6·0)% *versus* 47·7 (10·3)% (*P* = 0·001) respectively. On multivariate analysis, failure to consolidate MR with an auto‐SCT was associated with a significantly increased risk of relapse [hazard ratio (HR) 4·91, 95% confidence interval (CI) 1·56–15·41; *P* = 0·006]. MR induction with ATO‐based regimens followed by an auto‐SCT in children and young adults with relapsed APL who were treated with front‐line ATO‐based regimens was associated with excellent long‐term survival.

## Introduction

Acute promyelocytic leukaemia (APL) is a relatively rare subtype of acute myelogenous leukaemia (AML) that occurs in 8–15% of all AMLs.^1^ Facilitated by an increased understanding of the molecular mechanisms of the disease and resistance, significant advances have been made in the management of APL over the last two decades. There has been a steady transition over the years to a non‐myelotoxic therapy consisting of arsenic trioxide (ATO) combined with all‐*trans* retinoic acid (ATRA).^2^ Optimal treatment of APL requires rapid initiation of ATO and/or ATRA therapy and intensive supportive care for APL‐specific complications, including bleeding disorders and APL differentiation syndrome.^1^ The widespread clinical employment of this combination of ATO + ATRA has reduced relapse from ~50% to <10% in adult patients with APL over the past two decades.^1^

Challenges remain in the management of patients with high‐risk disease at presentation and those with relapsed APL. Salvage therapies with combined ATRA and arsenic compound‐based regimens have been shown to induce a complete remission (CR) in 50–80% of patients with refractory or relapsed APL.^1^, ^3^ The available data for the management of relapsed APL are mostly in the context of relapse after conventional ATRA plus chemotherapy‐based regimens and in this setting the existing data suggests that an autologous stem cell transplant (auto‐SCT) in molecular remission (MR) is associated with better survival in comparison to ATO‐based maintenance therapy.^1^, ^4^, ^5^, ^6^, ^7^, ^8^

The standard of care for patients with APL relapsing after front‐line treatment with ATO‐based regimens remains to be defined. Reports on response and survival outcomes in patients who had received ATO as part of their up‐front therapy suggest that there is a high incidence of resistance to ATO and ATRA resulting in inferior response and survival^9^, ^10^, ^11^, ^12^; ~25% of relapsed APL treated with up‐front ATO were noted to have promyelocytic leukaemia (*PML*) gene mutations, and survival in these patients was reported to be <30%, this is in contrast to experience at most other centres. In our single‐centre experience, failure to achieve MR with ATO‐based regimens in relapsed APL is very rare.^13^ Further, in an international multicentre study that our institution was involved in, evaluating the mutational landscape of newly diagnosed and relapsed APL, it was noted that the occurrence of mutations that resulted in primary or secondary ATO resistance was also extremely rare.^14^

We present a retrospective analysis of our experience in the management of relapse in patients with APL treated up‐front with ATO‐based regimens.

## Patients and methods

**Fig 1:**
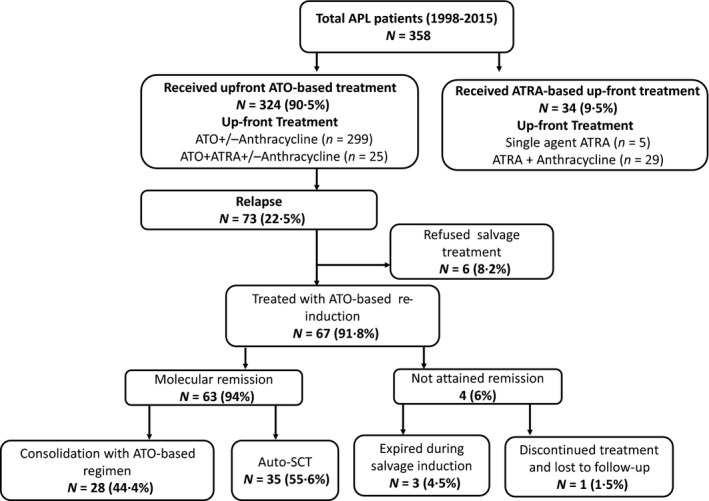
Flow chart of the patients treated. Of the 67 patients with relapsed acute promyelocytic leukaemia (APL) treated with arsenic trioxide (ATO)‐based re‐induction therapy, 63 (94%) achieved molecular remission; 35 (55·6%) underwent autologous stem cell transplantation (Auto‐SCT) and 28 (44·4%) were consolidated with ATO‐based maintenance chemotherapy. ATRA, all‐*trans*retinoic acid.

All consecutive patients with relapsed APL who received up‐front ATO‐based therapy, who were diagnosed and treated between January 1998 and December 2015, were included in the analysis. In addition to patients who were initially treated for a diagnosis of APL at our centre, we also included patients who were initially treated at other centres and referred to us at relapse provided adequate details of their initial treatment was available (summarised in Fig 1). Up‐front therapy in these patients included single‐agent ATO or a combination of ATO and ATRA with or without anthracycline in induction and consolidation administered in a risk‐adjusted manner and was followed by ATO‐based maintenance therapy. Until December 2014, we used the single‐agent ATO‐based regimen, the details of the protocol and the early and long‐term data have been reported previously by our group.^15^, ^16^ From January 2015, a combined ATO and ATRA regimen was used along with anthracylines for high‐risk APL (Figure S1 summarises the protocols that were used). Minimal residual disease (MRD) was monitored by qualitative periphral blood reverse transcription polymerase chain reaction (RT‐PCR), as previously reported by us.^17^ In brief, RT‐PCR analysis for *PML*‐retinoic acid receptor alpha (*RARA*)_fusion transcripts with a sensitivity of 10^−4^ was done using Europe Against Cancer programme protocols, the assays were run on an ABI PRISM 7000 DNA Sequence Detection System (Thermo Fisher Scientific, Inc., Waltham, MA, USA). The MRD assay was done once every 3 months for 2 years and up to 5 years for high‐risk cases. This retrospective study protocol was reviewed and approved by the Institutional Review Board (IRB 12120; dated 26 June 2019).

### Treatment of relapsed APL

#### Salvage chemotherapy

All relapses were confirmed by morphology and molecular investigations. Only patients who were positive for t(15;17) by karyotyping, fluorescence *in situ* hybridisation (FISH), or RT‐PCR were included in this analysis. A diagnostic lumbar puncture was done on all patients at the time of suspected relapse evaluation with adequate blood product cover as required. After the diagnosis of relapse, all patients received induction treatment with the ATO‐based regimen, either alone or in combination with ATRA and anthracycline as per the treating physician’s discretion.^13^, ^16^ ATO was adminsitered, as we always do, at a dose of 0·15 mg/kg for paediatric patients and those whose weight is <45 kg to a maximum dose of 10 mg/day. All adults and those weighing ≥45 kg received a fixed dose of 10 mg/day. Since 2013, patients who had relapsed APL after front‐line exposure to ATO were enrolled in an ongoing clinical trial that added bortezomib to the salvage regimen.^18^ Salvage therapy at relapse consisted of induction followed 4 weeks later by consolidation, usually with the same agents used in induction (illustrated in Figure S2), all salvage regimens were centred on the use of ATO in combination with other drugs. Table I summarises the different types of salvage regimens that were utilised. All patients who achieved a MR at the end consolidation were offered an auto‐SCT. Methods used for molecular monitoring were as reported previously by us.^17^ For those who opted not to undergo an auto‐SCT, usually due to financial constraints, a maintenance regimen of ATO‐ or ATO + ATRA‐based regimens at standard doses were administered for 10 days a month for 6 months. Patients who had central nervous system (CNS) involvement at relapse also received triple intrathecal (2 doses/week × 6 doses) concomitant cranial radiotherapy (2.4 Gy), along with the salvage systemic therapy. For those with isolated CNS relapse post‐remission induction, they received one more course of systemic consolidation therapy followed by maintenance therapy and they were not offered an auto‐SCT.

**Table I:** Patients’ baseline characteristics.

Variable	Value
Age, years, median (range)	28 (4–54)
≤12 years, *n* (%)	13 (19)
Gender, *n* (%)	
Male	44 (65·7)
Female	23 (34·3)
Time to relapse from diagnosis, months, median (range)	19·6 (5·9–128·4)
≤2 years, *n* (%)	41 (61)
>2 years, *n* (%)	26 (39)
Sites of relapse, *n* (%)	
Isolated molecular	1 (1·5)
Isolated CNS	6 (9·0)
Marrow + CNS	13 (19·4)
Isolated marrow	47 (70·1)
Salvage therapy at relapse, *n* (%)	
ATO	5 (7·5)
ATO + anthracycline	1 (1·5)
ATO + bortezomib	3 (4·5)
ATO + ATRA	10 (14·9)
ATO + ATRA + anthracycline	26 (38·8)
ATO + ATRA + anthracycline + bortezomib	22 (32·8)
Response to salvage therapy, *n* (%)	
Molecular remission	63 (94·0)
Discontinued treatment	1 (1·5)
Expired	3 (4·5)
Consolidation at CR2 (*n* = 63), *n* (%)	
Auto‐SCT	35 (55·6)
ATO‐based maintenance	28 (44·4)
Time to auto‐SCT, months, median (range) (*n* = 35)	5·8 (3·8–32·2)
ATO‐based maintenance (*n* = 28), *n* (%)	
ATO alone	6 (21·4)
ATO + ATRA	22 (78·6)
Status at last follow‐up, *n* (%)	
Auto‐SCT Group (*n* = 35)	
Continued in CR2	32 (91·4)
Second relapse and expired	2 (5·7)
in CR3	1 (2·9)
ATO‐based maintenance Group (*n* = 28), *n* (%)	
Continued in CR2	17 (60·7)
Second relapse and expired	8 (28·6)
in CR3	3 (10·7)

ATO, arsenic trioxide; ATRA, all‐*trans* retinoic acid; auto‐SCT, autologous stem cell transplant; CNS, central nervous system; CR, complete remission.

#### Auto‐SCT

All patients scheduled for auto‐SCT underwent peripheral blood stem cell (PBSC) collection after chemo‐mobilisation with granulocyte colony‐stimulating factor (G‐CSF) administered at a dose of 10 μg/kg/day for 4 days. The targeted CD34 stem cell dose was 5 × 10^6^/kg, and a minimum dose of 2 × 10^6^/kg was required to proceed with the transplant. The conditioning regimen consisted of oral busulfan (Bu) at a dose of 16 mg/kg administered over 4 days, followed by cyclophosphamide (Cy) administered intravenously at a dose of 120 mg/kg over 2 days. The mobilised and cryopreserved PBSC product was thawed and rapidly infused a day after the completion of the conditioning regimen. All patients were started on G‐CSF (5 μg/kg/day) from day 7 of PBSC infusion and continued until neutrophil engraftment occurred. After auto‐SCT, maintenance intrathecal methotrexate was administered once a month for 6 months.

### Definition of outcomes

Achievement of CR required that a patient have no clinical evidence of APL, an absolute neutrophil count (ANC) of >1·0 × 10^9^/l, and unsupported platelet count of >100 × 10^9^/l, as well as bone marrow (BM) showing normo‐cellularity to moderate hypo‐cellularity, with <5% blasts plus pro‐myelocytes. An exception was made in patients with an ANC of <1.0 × 10^9^/l who achieved an unsupported platelet count of >100 × 10^9^/l for >2 weeks with no evidence of residual disease, as defined previously.^4^, ^16^ Molecular relapse was defined as two consecutive positive RT‐PCRs obtained 1 month apart after achieving MR. Overall survival (OS) and event‐free survival (EFS) were defined as the time from initiation of treatment to death due to any cause and the time from the start of treatment to relapse or death due to any reason respectively.

### Statistical analysis

For descriptive statistics, the median and range were used as appropriate, while for categorical variables, number and proportion were used. For the association between two categorical variables, a chi‐square test/Fisher’s exact test was used. The cumulative probability of survival was estimated using the Kaplan–Meier method for OS and EFS, and a log‐rank test was used to compare two or more survival curves. The study variables that were significant at <0·05 levels in a univariate analysis alone were included in a multivariate Cox proportional hazards model. The model assumption was verified using log–log S (t) plots and Global test. A *P* < 0·05 was considered as statistically significant. All statistical analyses were performed using the Statistical Package for the Social Sciences (SPSS®), version 21·0 (IBM Corp., Armonk, NY, USA).

## Results

### Baseline patient characteristics

Out of the total 358 patients diagnosed with APL during the study period, 104 (29%) patients relapsed; 73 (70%) had received up‐front ATO‐based treatment (70 had received up‐front ATO ± anthracycline and three received up‐front ATO + ATRA ± anthracyline), six (8·2%) of these patients abandoned further treatment, while the remaining 67 (91·8%) patients who received an ATO‐based salvage regimen were included in this analysis (summarised in Fig 1). The six patients who abandoned therapy were all in poor general condition with sepsis at admission and they were discharged at request on supportive measures. None of them received any disease‐specific treatment and on telephone follow‐up they had all died over the next few days. During this period, there were a total of 1659 patients diagnosed to have AML other than APL at our centre. The median (range) age of the patients with APL and AML was 30 (3–75) and 36 (0–85) years respectively (Figure S3).

The baseline and treatment characteristics of the 67 patients analysed are summarised in Table I. The median (range) age of the patients was 28 (4–54) years, 13 (19%) were in the paediatric age group (≤12 years) and 44 (66%) were males. The median (range) time to relapse was 19·6(5·9–128·4) months. The majority of patients (61%) relapsed within 2 years of initial diagnosis, 19 (28%) between 2 and 5 years, and four (6%) relapsed after 5 years of diagnosis and one of these (a 9‐year‐old child) had relapsed after 10 years. Except for one patient who only had evidence of a molecular relapse; the remaining patients had a morphologically documented relapse. The majority of the morphological relapses were in the BM 47 (70·1%). There were six (9%) isolated CNS relapses and 13 (19·4%) having both medullary and CNS disease at the time of diagnosis of relapse.

### Post‐relapse salvage therapy

The majority of patients (71·6%) received a combination of ATO + ATRA + anthracycline at relapse. One patient opted to discontinue treatment while on induction therapy and was discharged at request against medical advice. Three patients (4·5%) died during induction therapy (two had presented with severe neutropenic sepsis, and one at presentation had an intracranial bleed). The remaining 63 patients (94%) achieved haematological remission, and all these patients achieved MR, as previously defined by us,^17^ after completion of their first consolidation therapy.

### Auto‐SCT

As per standard recommendations, all patients who achieved MR after salvage therapy were offered an auto‐SCT; however, only 35 (56%) patients who had financial resources opted for an auto‐SCT. The median (range) CD34 cell dose was 8·3 (2·5–24·3) × 10^6^/kg; 13/35 (37·1%) required a second‐day harvest to achieve target stem cell dose. The entire harvested cell product was infused after completion of the conditioning regimen. All the patients had documented neutrophil and platelet engraftment at a median (range) of 11 (9–13) and 17 (9–33) days respectively.

Although febrile neutropenia was documented in 88·6% of the patients, only six (17%) had documented bacteraemia. None of the patients developed a fungal infection after transplant. Mucositis was documented in 32 (91·4%) patients. Among the 35 patients who underwent auto‐SCT, there was no transplant‐related mortality. Maintenance intrathecal chemotherapy after auto‐SCT (monthly methotrexate × 6 months) starting from 3 months after auto‐SCT was offered to all; however, only 28 (80%) received intrathecal therapy as per the planned schedule. Three (8·6%) patients in the auto‐SCT group had a second relapse at a median (range) of 10 (4·6–21·9) months after auto‐SCT. One of these relapsed patients underwent allogeneic SCT and is in CR3 at the last follow up visit (>10 years from initial diagnosis and 82 months after the second relapse). The other two died of sepsis soon after the second relapse.

### ATO‐based maintenance therapy

Of the 63 patients in CR2, 28 (44%) opted against auto‐SCT due to social and financial constraints and were given ATO‐based maintenance therapy for 10 days a month × 6 months. Those with CNS involvement at relapse also received monthly intrathecal methotrexate for 6 months. The majority (78·6%) received an ATO + ATRA‐based regimen as maintenance, while six patients received only ATO as maintenance therapy (four of them had isolated CNS relapse and two had isolated BM relapse). The maintenance regimen was administered on an outpatient basis, and the Eastern Cooperative Oncology Group (ECOG) Performance Score during this period was 0/1 in all cases. There was no evidence of significant toxicity that warranted discontinuation of maintenance in any patient, and all patients completed the scheduled maintenance regimen. Of the 28 patients on ATO‐based maintenance therapy, 11 (39·3%) had a second relapse at a median (range) of 12·3 (5·4–16·2) months, and one died during follow up (cause unknown). Three of the 11 relapsed patients subsequently underwent auto‐SCT after salvage therapy (ATO‐based regimen) and achieved a MR and remain in continuous CR3 at the last follow‐up visit (at 48, 55 and 67 months from initial diagnosis and 9, 21 and 30 months from the second relapse). Characteristics of patients who received an auto‐SCT *versus* those that received maintenance with ATO‐based therapy are compared in Table II.

**Table II:** Comparison of patient characteristics assigned to auto‐SCT *versus* ATO‐based chemotherapy.

Variable	Auto‐SCT (*n* = 35)	ATO‐based chemotherapy (*n* = 28)	*P*
Age, years, median (range)	28 (4–53)	26 (6–54)	0·961
Gender, *n* (%)			
Male	26 (74·3)	15 (53·6)	0·087
Female	9 (25·7)	13 (46·4)	
Time to relapse from diagnosis, *n* (%)			
≤2 years	19 (54·3)	19 (67·9)	0·274
>2 years	16 (45·7)	9 (32·1)	
Sites of relapse, *n* (%)			
Marrow + CNS	7 (20)	5 (17·9)	0·017
Isolated marrow	27 (77·1)	17 (60·7)	
Isolated CNS	–	6 (21·4)	
Isolated molecular	1 (2·9)	0 (0)	
Salvage therapy at relapse, *n* (%)			
ATO based*	4 (5·7)	14 (28·6)	0·000
ATO + ATRA + anth ± bortezomib†	31 (94·3)	14 (71·4)	
Post‐consolidation relapse, *n* (%)			
Yes	4 (11·4)	12 (42·9)	0·004
No	31 (88·6)	16 (57·1)	

anth, anthracycline; ATO, arsenic trioxide; ATRA, all‐*trans* retinoic acid; CNS, central nervous system; auto‐SCT, autologous stem cell transplant.

* ATO‐based chemotherapy included ATO alone = 4, ATO + anthracycline = 1, ATO + bortezomib = 3, ATO + ATRA = 10.

† 22 patients received bortezomib along with ATO + ATRA + anthracycline.

### Survival

**Fig 2:**
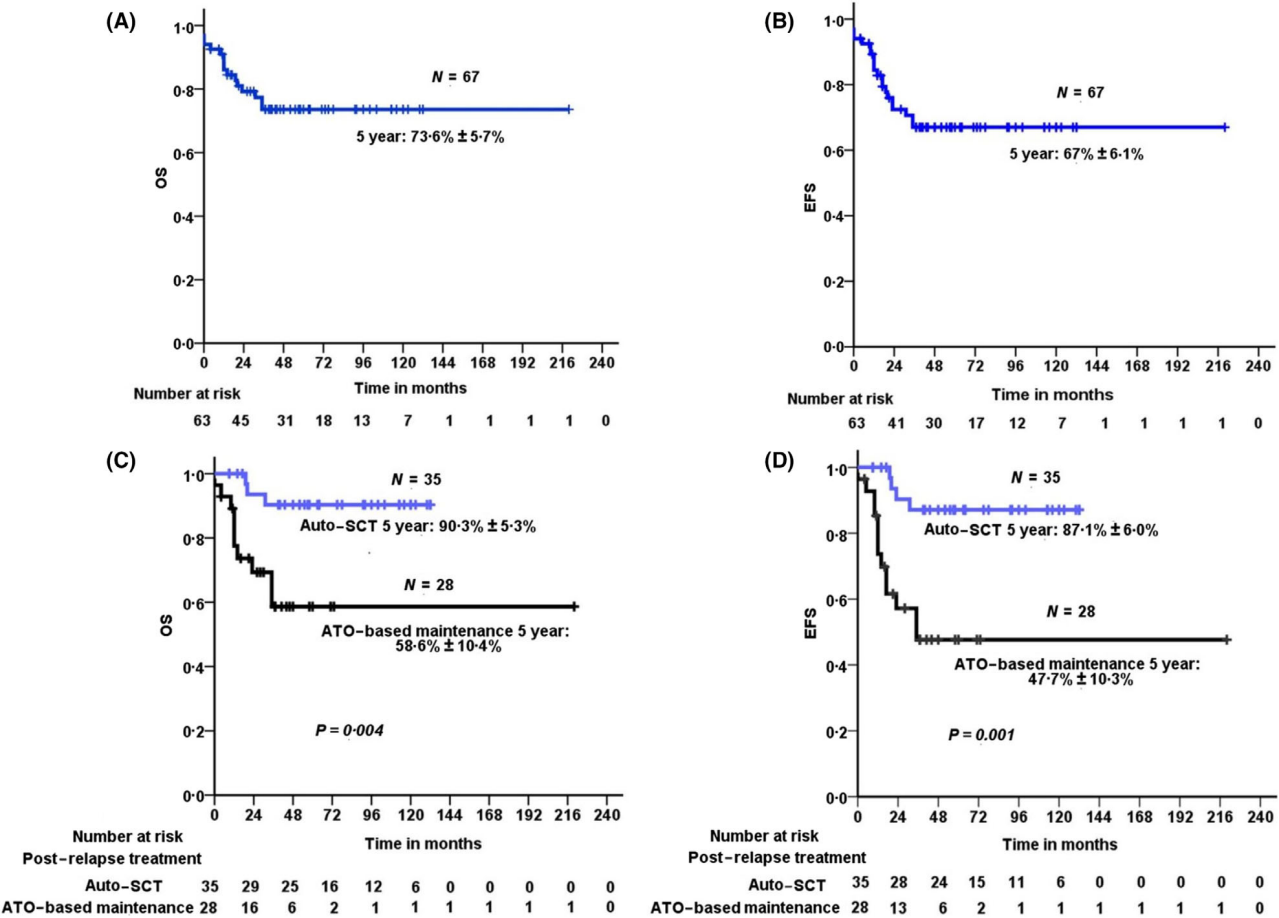
Kaplan–Meier estimate of overall survival (OS) and event‐free survival (EFS) of patients with relapsed acute promyelocytic leukaemia treated with up‐front arsenic trioxide (ATO)‐based regimens. The mean (SD) 5‐year (A) OS and (B) EFS for the whole cohort (*N* = 67) was 73·6 (5·7)% and 67 (6·1)% respectively. The mean (SD) 5‐year (C) OS and (D) EFS of patients consolidated with autologous stem cell transplant (SCT) (*n* = 35) *versus* A TO‐based chemotherapy maintenance (*n* = 28) was 90·3 (5·3) % *versus* 58·6 (10·4) % (*P* = 0·004) and 87·1 (6·0) % *versus* 47·4 (10·3) % (*P* = 0·001) respectively. Auto‐SCT, autologous stem cell transplantation. [Colour figure can be viewed at wileyonlinelibrary.com]

With an actuarial median (range) follow up of 43 (0–220) months, the mean (SD) 5‐year Kaplan–Meier estimates of OS and EFS of the whole cohort (Fig 2A, B) were 73·6 (5·7)% and 67 (6·1)% respectively. The mean (SD) 5‐year Kaplan–Meier estimates of OS and EFS of those who underwent auto‐SCT were 90·3 (5·3)% and 87·1 (6·0)% respectively. In comparison, similar estimates for those on ATO‐based maintenance therapy were significantly lower at a mean (SD) of 58·6 (10·4)% and 47·4 (10·3)% respectively (Fig 2C, D). The survival analysis was repeated using a ‘landmark method’ from the time of SCT in one group and from starting maintenance therapy in the other, which were not significantly different in the two groups from the date of diagnosis of relapse. There was no significant variation from the survival data presented above nor did it change the data of the multivariate analysis signifcantly (Table SI), ruling out the effect of an immortal time bias in the analysis.

### Impact on clinical outcome of additional factors

On analysing the factors impacting EFS using Cox regression analysis, those who received only ATO‐based maintenance therapy instead of auto‐SCT consolidation after having achieved MR had an increased risk of relapse, both on univariate and multivariate analysis [hazard ratio (HR) 5·73, 95% confidence interval (CI) 1·86–17·66, *P* = 0·002; and HR 4·91, 95% CI 1·56–15·41, *P* 0·006 respectively) compared to those that received auto‐SCT (Table III). On univariate analyses, no significant impact of age, total white blood cell (WBC) count at relapse, site of relapse, early *versus* late relapse (≤2 vs. >2 years) or adult *versus* paediatric patients (≤12 vs. >12 years) on survival were noted on comparing these two groups (Table SII also shows the comparison between those aged ≤18 vs. >18 years). All the events in the group that did not undergo auto‐SCT were due to relapses and there were no deaths due to regimen‐related toxicity or inadequate medical support, reflecting the inadequacy of the consolidation therapy in these patients.

**Table III:** Adjusted Cox regression analysis.

Variable	Unadjusted analysis		Adjusted analysis	
	HR (95% CI)	*P*	HR (95% CI)	*P*
Post‐relapse treatment				
Auto‐SCT	1·00		1·00	
ATO‐based chemotherapy	5·73 (1·86–17·66)	0·002	4·91 (1·56–15·41)	0·006
Duration of CR1	0·998 (0·997–0·999)	0·025	0·999 (0·997–1·00)	0·070
Total WBC at relapse	1·01 (0·99–1·03)	0·162	–	–
Age	1·003 (0·97–1·04)	0·853	–	–
Salvage therapy at relapse*				
ATO based	1·00		–	–
ATRA + anth ± bortezomib	0·81 (0·30–2·18)	0·673	–	–

anth, anthracycline; ATO, arsenic trioxide; ATRA, all‐*trans* retinoic acid; CNS, central nervous system; auto‐SCT, autologous stem cell transplant.

* There were 25 patients who received bortezomib (three along with ATO and 22 along with ATO + ATRA + anth) as part of salvage chemotherapy at relapse].

Mutation analysis was not done routinely as part of our clinical service. However, ~50% of the patients in this cohort had undergone *PML* mutation analysis as part of two previously reported studies,^13^, ^14^ and 15% of relapsed patients who were tested were noted to have *PML‐B2* domain mutations. None of the mutations that we detected were associated with secondary ATO resistance, all those with mutations achieved MR, and none of our relapsed patients had the *PML‐B2* A216V mutation.^14^

## Discussion

The standard of care for managing patients with relapsed APL when ATO has been used as up‐front therapy has not yet been established. In the recently published European LeukemiaNet (ELN) guidelines, a new recommendation was to consider using an ATRA + chemotherapy‐based regimen unless the relapse happened >2 years of CR1^1^; this was based on an expert consensus and not on any large dataset or clinical experience (Level IVC recommendation). We have previously reported that *PML‐RARA* mutations that contribute to secondary ATO resistance were infrequent and that with a rare exception, most patients achieve complete MR with an ATO‐based re‐induction for relapse and even after multiple relapses.^13^, ^14^ Considering the proven efficacy of ATO alone or combination with ATRA/chemotherapy in newly diagnosed APL, in relapsed APL post‐ATRA plus chemotherapy regimens and based on our experience in relapsed APL treated with up‐front ATO, it would be reasonable to consider ATO‐based combination therapies for patients with relapsed APL treated with up‐front ATO‐based regimens as well.^18^, ^19^, ^20^ Our present data do not suggest that either early or late relapses after up‐front ATO‐based therapy has an impact on the ability of repeat ATO‐based regimens to induce remission or survival in these patients. The theoretical concerns of ATO resistance^10^, ^11^ in patients who relapse after up‐front ATO‐based regimens do not appear to be a relevant concern based on our present data and in communications with other centres in the clinic.

Although auto‐SCT is offered to all patients with relapsed APL who achieve CR2 at our centre, due to financial constraints, only a proportion of these patients can afford this approach as previously reported by us^4^; hence, a substantial proportion of patients opt to continue ATO maintenance without an auto‐SCT. As a result, we were in a unique position to do this retrospective comparative analysis of patients with relapsed APL who underwent consolidation therapy with either auto‐SCT or a combination of ATO and ATRA maintenance therapy.

Our experience, as summarised in the present analysis, demonstrated that ATO‐based combination therapy was as effective in re‐inducing complete MR in patients with relapsed APL with previous exposure to ATO‐based therapy in both the paediatric and the adult population, as well as in early (≤2 years of CR1) and late relapses. However, our present data would suggest that for sustained remission, these patients should be consolidated with an auto‐SCT after they have achieved a MR. The clinical outcomes with this approach are associated with excellent results, as reported here [mean (SD) 5‐year OS and EFS of 90·3 (5·3)% and 87·1 (6·0)% respectively], and is similar to the observation in patients with relapsed APL treated with up‐front ATRA‐based regimens.^4^, ^5^, ^6^, ^7^, ^20^, ^21^ While maintenance with an ATO‐based regimen is well tolerated, the risk of subsequent relapse is very high (39·3%) compared to that seen in the group that received an auto‐SCT consolidation (8·6%). Additionally, in our experience, cases that relapsed after ATO‐based maintenance therapy were difficult to salvage, with a median time to death from such relapses of <1 month.

While the number of paediatric patients in the present study was small, the available data would suggest that the results of salvage with an ATO‐based regimen followed by consolidation with auto‐SCT are similar to those seen in adults and to several previous reports.^7^ As observed in adults, there was no transplant‐related mortality in children who underwent auto‐SCT. Moreover, with a median (range) follow up of 43 (0–220) months, no long‐term complications were observed in these children.

A limitation of the present study is the retrospective nature of the study and the relatively small numbers evaluated in a single institution setting. However, considering the relatively small number of relapses in patients with current treatment strategies of newly diagnosed APL, it is unlikely that a large Phase III trial would ever be done to address this question. The relatively younger population in the present study is not due to selection bias (all consecutive cases were included and accounted for), but a reflection of the population pyramid in low‐ and middle‐income (LMIC) countries, such as ours, with a significantly lower median age of diagnosis of patients with APL and AML in LMIC tertiary centres as illustrated in Figure S2. Extrapolating, these data to older patients would be difficult due to the complete absence of cases aged >55 years in our present analysis.

Cumulatively, our observation is that in relapse after up‐front ATO‐based therapy in young adults and paediatric patients an ATO‐based re‐induction is appropriate, well‐tolerated and associated with good response rates immaterial of whether the relapse happened early (<2 years) or late. Auto‐SCT in this group of patients as consolidation has a clear survival advantage over ATO‐based maintenance therapy alone and could be considered the standard of care in this group.

## Conflict of interest

None.

## Author contributions

N.A. Fouzia: performed research, designed study, analysed data and wrote the paper. V. Sharma, U.P. Kulkarni, A. Korula, A.J. Devasia, S.C. Nair, A. Abraham and B. George: performed research and analysed data. S. Ganesan, H.K. Palani, N. Balasundaram, S. David, N. Beryl Janet and P. Balasubramanian: performed research, performed molecular tests, and analysed data. T. Mani and J. Lakshmanan: statistical analysis of data. V. Mathews: performed research, designed study, clinical data accrual, analysed data, and wrote the paper.

## Supporting information


**Figure S1.** Summary of up‐front ATO‐based regimens that were used.
**Figure S2.** Salvage chemotherapy protocol for relapsed APL.
**Figure S3.** Age distribution of patients with AML and APL diagnosed during the period of this study at our centre (1998–2015).
**Table SI.** Adjusted Cox regression analysis (done using date of SCT and date of starting maintenance therapy).
**Table SII.** Comparison of patient characteristics based on age ≤18 *versus* >18 years.
