# Supplementary material for: Management of relapse in acute promyelocytic leukaemia treated with up‐front arsenic trioxide‐based regimens
Source: Br J Haematol. 2020 Nov 20;192(2):292–9. doi: 10.1111/bjh.17221 (PMC7894296; doi:10.1111/bjh.17221)
Supplement: Supplementary file 1 — Figure S1. Summary of up‐front ATO‐based regimens that were used. Figure S2. Salvage chemotherapy protocol for relapsed APL. Figure S3. Age distribution of patients with AML and APL diagnosed during the period of this study at our centre (1998–2015). Table SI. Adjusted Cox regression analysis (done using date of SCT and date of starting maintenance therapy). Table SII. Comparison of patient characteristics based on age ≤18 versus >18 years. [file BJH-192-292-s001.docx]

# Management of Relapse in Acute Promyelocytic Leukaemia Treated with Upfront Arsenic Trioxide Based Regimens

Fouzia NA^1^, Vibhor Sharma^1^, Saravanan Ganesan^1^, Hamenth Kumar Palani^1^, Nithya Balasundaram^1^, Sachin David^1^, Uday P Kulkarni^1^, Anu Korula^1^, Anup J Devasia^1^,Sharon Lionel^1^, Sukesh C. Nair^2^, Nancy Beryl Janet^1^, Aby Abraham^1^,  Thenmozhi Mani^3^, Jeyaseelan Lakshmanan^3^, Poonkuzhali Balasubramanian^1^, Biju George^1^, Vikram Mathews^1^

^1^ Department of Haematology, Christian Medical College, Vellore, India

^2^Department of Immunohaematology and Transfusion Medicine, Christian Medical College, Vellore, India.

^3^ Department of Biostatistics, Christian Medical College, Vellore, India.

**Supplementary figure S1: Summary of upfront ATO based regimens that were used**

1. Single agent ATO based regimen([1](#_ENREF_1)), until December 2014

Single agent ATO until CR (max 60 days). 4 weeks break followed 4 weeks consolidation, second 4 week break and then 10 days a month for 6 months.

Hydroxyurea used to control leucocytosis in induction. Anthracyclines permitted in cases with rapid rise in leucocyte count after starting treatment and for differentiation syndrome, as defined in detail by us in an earlier pblication([1](#_ENREF_1)).

1. From 2015, ATRA was added to the above regimen for all cases. In high risk cases anthracycline was added in induction and to the first consolidation cycle. A second consolidation cycle was introduced with ATO+ATRA and then onto maintenance as above.

**Supplementary figure S2: Salvage chemotherapy protocol for relapsed APL**

***Induction**

Inj. ATO 10 mg / day (<45 kg: 0.15mg/kg/day) x minimum 6 weeks

Cap. ATRA 45mg / m2 / day in 2 divided doses ] x maximum 60 days

Inj. Mitoxantrone 10 mg / m2 / day ] Day 1 and 2

*** Consolidation**

Injection ATO 10 mg / day (<45 kg 0.15mg/kg/day) ] x 28 days

Cap ATRA 45mg / m2 / day in two divided doses ] x 28 days

*** Maintenance**

Inj. ATO 10 mg / day (<45 kg – 0.15 mg/kg) 10 days / month x 6 months

Cap ATRA 45mg / m2/ day in two divided dose 10 days/month

Intrathecal Methotrexate 12.5 mg on day 1 of each cycle

**Supplementary figure S3: Age distribution of AML and APL patients diagnosed during the period of this study at our centre (1998 – 2015)**

**Newly diagnosed AML n= 1659**

**
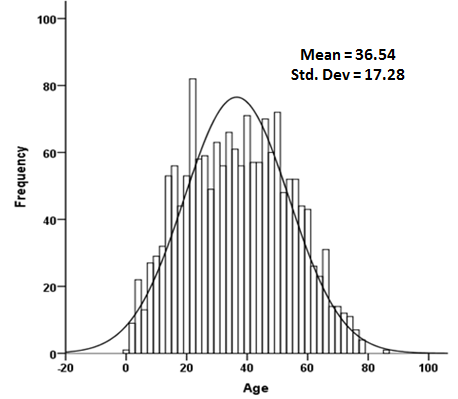
**

**Newly diagnosed APL n = 358**


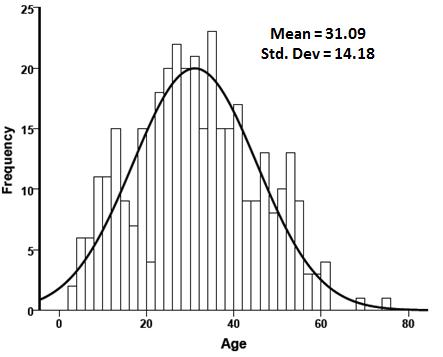


**Address immortal time bias in analysis:** We repeated the analysis as shown below using a ‘landmark method’ where the start of analysis was either the date of SCT or the date of starting maintenance therapy (which are comparable in the two group with no statistically significant difference from date of diagnosis of relapse and the above time points between the two groups). There was no significant variation in survival curves between the two groups to what we have reported with this variation and the Cox model with this entry criteria method did not show any significant difference in comparison to the results we have reported. Based on this we do not feel there as any evidence of an immortal time bias in our analysis

**Table 1: Adjusted Cox regression analysis (done using date of SCT and date of starting maintenance therapy)**

|  | **Unadjusted Analysis** | | | **Adjusted Analysis** | | |
| --- | --- | --- | --- | --- | --- | --- |
| **Variables** | **HR** | **95% CI for HR** | **P value** | **HR** | **95% CI for HR** | **P value** |
| **Post Relapse Treatment**  Autologous SCT  ATO based chemotherapy | 1.00  5.34 | (1.73 – 16.46) | 0.003 | 1.00  4.49 | (1.43 – 14.12) | 0.010 |
| Duration of CR1 | 0.998 | (0.996 – 0.999) | 0.023 | 0.999 | (0.997 – 1.00) | 0.065 |
| Total WBC at relapse | 1.01 | (0.99 – 1.03) | 0.170 | - | - | - |
| Age | 1.004 | (0.97 – 1.04) | 0.845 | - | - | - |
| **Salvage therapy at relapse**  ATO based  ATRA+Anth**±**Bortezomib | 1.00  0.83 | (0.31 – 2.26) | 0.723 | - | - | - |

**Supplementary Table 2: Comparison of patient characteristics based on age ≤=18yrs versus >18 yrs**

| **Variables** | **Age ≤ 18 yrs**  **(n=20)** | **Age >18 yrs**  **(n=43)** | **P-value** |
| --- | --- | --- | --- |
|  | N (%)/ Median (range) | N (%)/ Median (range) |  |
| **Gender**  Male  Female | 14(70.0)  6(30.0) | 27(62.8)  16(37.2) | 0.576 |
| **Time of relapse from diagnosis**  **≤ 18**6 months  >18 months | 9(45.0)  11(55.0) | 18(41.9)  25(58.1) | 0.815 |
| **Time of relapse from diagnosis**  **≤**  2 yrs  >2 yrs | 11(55.0)  9(45.0) | 27(62.8)  16(37.2) | 0.556 |
| **Sites of relapse**  Marrow + CNS  Isolated marrow  Isolated CNS  Isolated molecular | 3(15.0)  17(85.0)  0(0)  0(0) | 9(20.9)  27(62.8)  6(14.0)  1(2.3) | 0.219 |
| **Salvage therapy at relapse**  ATO based*  ATO+ATRA+Anth**±**Bortezomib | 3(15.0)  17(85.0) | 15(34.9)  28(65.1) | 0.104 |
| **Consolidation at CR**  Auto-SCT  Chemotherapy based maintenance | 11(55.0)  9(45.0) | 24(55.8)  19(44.2) | 0.952) |
| **Survival**  OS  EFS | 74.3±10.0  60.0±11.0 | 78.6±6.8  75.6±7.2 | 0.739  0.203 |

1. Mathews V, George B, Lakshmi KM, Viswabandya A, Bajel A, Balasubramanian P, et al. Single-agent arsenic trioxide in the treatment of newly diagnosed acute promyelocytic leukemia: durable remissions with minimal toxicity. Blood. 2006;107(7):2627-32.
